# Genome-Wide Analysis of *MADS-Box* Genes in Foxtail Millet (*Setaria italica* L.) and Functional Assessment of the Role of *SiMADS51* in the Drought Stress Response

**DOI:** 10.3389/fpls.2021.659474

**Published:** 2021-06-28

**Authors:** Wan Zhao, Li-Li Zhang, Zhao-Shi Xu, Liang Fu, Hong-Xi Pang, You-Zhi Ma, Dong-Hong Min

**Affiliations:** ^1^College of Agronomy, Northwest A&F University/State Key Laboratory of Crop Stress Biology for Arid Areas, Yangling, China; ^2^Institute of Crop Science, Chinese Academy of Agricultural Sciences/National Key Facility for Crop Gene Resources and Genetic Improvement, Key Laboratory of Biology and Genetic Improvement of Triticeae Crops, Ministry of Agriculture, Beijing, China; ^3^Xinxiang Academy of Agricultural Sciences of He’nan Province, Xinxiang, China

**Keywords:** MADS-box, phylogenetic analysis, expression profiling, abiotic stress responses, foxtail millet

## Abstract

MADS-box transcription factors play vital roles in multiple biological processes in plants. At present, a comprehensive investigation into the genome-wide identification and classification of *MADS-box* genes in foxtail millet (*Setaria italica* L.) has not been reported. In this study, we identified 72 *MADS-box* genes in the foxtail millet genome and give an overview of the phylogeny, chromosomal location, gene structures, and potential functions of the proteins encoded by these genes. We also found that the expression of 10 MIKC-type *MADS-box* genes was induced by abiotic stresses (PEG-6000 and NaCl) and exogenous hormones (ABA and GA), which suggests that these genes may play important regulatory roles in response to different stresses. Further studies showed that transgenic *Arabidopsis* and rice (*Oryza sativa* L.) plants overexpressing *SiMADS51* had reduced drought stress tolerance as revealed by lower survival rates and poorer growth performance under drought stress conditions, which demonstrated that *SiMADS51* is a negative regulator of drought stress tolerance in plants. Moreover, expression of some stress-related genes were down-regulated in the *SiMADS51*-overexpressing plants. The results of our study provide an overall picture of the *MADS-box* gene family in foxtail millet and establish a foundation for further research on the mechanisms of action of MADS-box proteins with respect to abiotic stresses.

## Introduction

Transcription factors play multiple roles over the entire life cycle of higher plants (Riechmann and Ratcliffe, 2000; Singh et al., 2002). In combination with *cis*-regulatory sequences, TFs activate or inhibit the expression of specific target genes in different tissues or cells, or in response to environmental conditions, and thus participate in the growth, development, morphogenesis, and biotic and abiotic stress responses process in plants (Liu et al., 1999). According to the PlantTFDB4.0^1^, 320,370 TFs from 165 plant species have been classified into 58 families (Jin et al., 2018). Among these families, MADS-box proteins comprise a large TF family and are ubiquitous in the plant kingdom (Alvarez-Buylla et al., 2008; Zhang et al., 2018). Based on the evolutionary relationships and sequence characterization, Alvarez-Buylla et al. (2008) classified MADS-box proteins into two major types, Type I and Type II, and they all have one thing in common; both types contain a MADS-box domain. Normally, Type I *MADS-box* genes in plants contain one or two exons, none or one intron, and the proteins encoded usually contain a highly conserved SRF-like MADS domain but lack a K domain (De Bodt et al., 2003b; Gramzow and Theißen, 2010). However, Type II *MADS-box* genes contain multiple introns and exons, and the corresponding proteins possess four domains; from the N to the C terminus these are a conserved MEF2-like MADS (M) domain, a less-well-conserved intervening (I) domain, a semi-conserved keratin-like (K) domain, and a C-terminal (C) domain and are thus also known as MIKC-type MADS-box proteins (De Bodt et al., 2003a; Kaufmann et al., 2005). The M domain is the most conserved region that enables the functions f or nuclear localization, dimerization, DNA binding, and accessory factor binding (McGonigle et al., 1996; Riechmann et al., 1996; Immink et al., 2002; Gramzow et al., 2010). The I domain contributes to MADS-box protein dimerization (Kaufmann et al., 2005), and the K domain plays an important role in the formation of higher-order complexes in addition to dimerization (Yang and Jack, 2004; Puranik et al., 2014; Theißen et al., 2016). The C domain is the most variable region and is responsible for transcriptional activation (Honma and Goto, 2001). On account of the diversity of the I and K domains, Type II MADS-box proteins are divided into two clades, MIKC^∗^ and MIKC^*c*^, in which MIKC^*c*^ proteins have a shorter I domain and a more conserved K domain than MIKC^∗^ proteins (Henschel et al., 2002). Furthermore, based on phylogenetic analysis, the MIKC^*c*^ proteins from angiosperms group into at least 14 distinct subclades; AG/STK (AGL11), AGL6, AGL12, AGL17, Bsister (GGM13), FLC, AP1 (SQUA), AP3 (DEF), PI (GLO), OsMADS32-like, SVP (StMADS11), SOC1(TM3), TM8, and SEP (Becker and Theißen, 2003; Zhao et al., 2006; Gramzow and Theißen, 2015). Within the same subgroup, *MADS-box* genes mostly share similar expression patterns and the proteins perform highly related functions.

As transcriptional regulators, *MADS-box* genes play crucial roles in ontogeny and signal transduction in higher plants (Schilling et al., 2018). At present, there are few published studies on Type I *MADS-box* genes, although the results of Masiero et al. (2011) indicate that Type I *MADS-box* genes are significant in plant reproductive development. Research has shown that the expression of Type I *MADS-box* genes in *Arabidopsis* can affect development of the female gametophyte, embryo, and endosperm (Day et al., 2008; Walia et al., 2009; Tiwari et al., 2010; Wuest et al., 2010). In contrast, studies on the plant-specific Type II *MADS-box* genes have been more thorough and extensive, and have shown that such genes play a broader regulatory role in plants. Firstly, the Type II *MADS-box* genes are closely related to flower development, and studies of the Type II MADS genes from several floral homeotic mutants in dicots have led to the establishment of the well-known ABCDE model for the determination of floral organs (Krizek and Fletcher, 2005). Additionally, multiple organs, including the roots, leaves, buds, embryos, and seeds were found to express Type II *MADS-box* genes, which provides further evidence for their diverse roles in plant development (Fornara et al., 2004; Gramzow and Theißen, 2010). For example, the expression of *MdDAM1*, a MIKC-type *MADS-box* gene from apple, was restricted to buds and could control growth cessation and bud dormancy (Moser et al., 2020). A recent study showed that under high temperature stress, AGAMOUS-LIKE67 (AGL67) could be combined with EARLY BOLTING IN SHORT DAY (EBS) to negatively control seed germination through the zinc-finger protein SOMNUS (SOM) in *Arabidopsis* (Li et al., 2020). With the increase in the number of research studies, a large number of MIKC-type genes have been identified from different species and shown to be involved in processes related to the stress response and hormone effects (Arora et al., 2007; Jia et al., 2018; Wang et al., 2018; Wei et al., 2018). For example, the MIKC-type MADS-box transcription factor SVP2 from kiwifruit (*Actinidia* sp.) was found to participate in ABA-mediated dehydration pathways by modulating expression of numerous target genes (Wu et al., 2018). Expression of the AGL2-like gene *ZMM7-L* from maize was affected by a variety of stresses, including cold, salt, drought, and exogenous ABA (Zhang Z. et al., 2012). In rice (*Oryza sativa* L.), the expression of *OsMADS61* (*OsMADS26*, an *AGL12* ortholog; Lee et al., 2008a), was enhanced in response to osmotic stress induced by D-mannitol, and further studies confirmed that overexpression of *OsMADS61* (OsMADS26; Khong et al., 2015) in rice had a negative impact on pathogen resistance and drought tolerance. Chen et al. (2019) found that the expression of an SEP/AGL2 subfamily *MADS-box* gene in pepper (*Capsicum annuum* L.) was induced by abiotic stresses (cold, heat, salt, and osmotic stress) and hormones (ABA, SA, and MeJA); in addition, the transgenic *CaMADS*-expressing plants were found to be more tolerant to low temperature, high salt, and mannitol treatments compared with WT plants (Chen et al., 2019). Also, more MIKC-type *MADS-box* genes associated with stress resistance have been identified in other species including wheat (*Triticum aestivum* L.), sheepgrass (*Leymus chinensis*), *Brassica rapa*, and tomato (*Solanum lycopersicum*) (Gopal et al., 2015; Guo et al., 2016; Jia et al., 2018; Schilling et al., 2020). These studies on the role of MIKC-type *MADS-box* genes on stress resistance provide crucial resources for potential use in plant breeding and crop improvement (Boden and Østergaard, 2019). At present, genome-wide analyses and functional characterization of MADS-box proteins have been conducted in many organisms, but there are few reports describing *MADS-box* genes in foxtail millet.

In this study, we identified a total of 72 *MADS-box* genes (29 Type I and 43 Type II) from the foxtail millet genome and we performed a comprehensive analysis of these genes. Ten MIKC-type *MADS-box* genes from different clades were shown to be induced by various abiotic stresses (drought and salinity) and exogenous application of hormones (ABA and GA). The *SiMADS51* genes, which belongs to the AGL12 subgroup, was isolated from cDNA of the foxtail millet cultivar ‘Yugu 1.’ The expression of *SiMADS51* was induced by PEG-6000, NaCl, ABA, and GA treatments. Further studies showed that overexpression of *SiMADS51* reduced the drought resistance of transgenic *Arabidopsis* and rice plants. These results will helpful in understanding the evolutionarily relationships, gene structures, and biological functions of the MADS-box TFs in foxtail millet and will establish a foundation for elucidating the drought resistance mechanism of *SiMADS51* gene.

## Materials and Methods

### Sequence Acquisition and Identification of *MADS-Box* Genes in Foxtail Millet

Hidden Markov Model (HMM) were employed to identify the *MADS-box* genes from foxtail millet (*Setaria italica* L.) genome. The implementation details were carried out as described by He et al. with some revisions (He et al., 2019). *Setaria italic* protein sequences were downloaded from the Phytozome V12.1 website^2^ to build a local protein database (Goodstein et al., 2012). The HMM profile of the SRF-TF domain (PF00319) was obtained from the Pfam database^3^. To acquire the foxtail millet *MADS-box* genes, the HMM profile were used to search against the local protein database by HMMER3 software with *E*-value < e^–5^ (Eddy and Pearson, 2011). Subsequently, all candidate proteins were checked for containing MADS domain by submitting them as search queries to the National Center for Biotechnology Information Conserved Domain Database (NCBI-CDD, Lu et al., 2019) and SMART (Ivica and Peer, 2017). MADS-box protein sequences of *Setaria viridis* were obtained by the same method above. MADS-box protein sequences from *Arabidopsis*, rice (*Oryza sativa* L.), and potato (*Solanum tuberosum*) were obtained from published studies (Parenicová et al., 2003; Arora et al., 2007; Gao et al., 2018). Ultimately, we acquired 72 *MADS-box* genes in foxtail millet, 76 in *Setaria viridis*, 75 in rice, 109 in *Arabidopsis* and 156 in potato and named them based on their chromosomal location order (Supplementary Table 1).

### Sequence Characteristics and Phylogenetic Analysis of MADS-Box Proteins in Foxtail Millet

Information regarding chromosomal distribution, predicted ORF length, number of amino acids, pfams, and introns of the foxtail millet *MADS-box* genes were downloaded from Phytozome V12.1. Isoelectric points (p*I*) and molecular weights (MW) were computed using the ProtParam tool on the ExPASy Server^4^. Full details are given in Table 1.

**TABLE 1:** The detailed information of 72 *MADS-box* genes identified in foxtail millet genome.

Gene Name	Transcript name	Chr	Pfam	Nucleotide length (bp)	Amino acid length (aa)	p*I*	MW (kDa)	Intron number	Subfamily
*SiMADS01*	Seita.1G003500.1	1	PF00319; PF01486	1425	474	8.73	53.18	7	SOC1(TM3)
*SiMADS02*	Seita.1G072200.1	1	PF00319; PF01486	750	249	6.14	28.11	5	Bsisiter
*SiMADS03*	Seita.1G077600.1	1	PF00319	966	321	5.94	34.22	0	M
*SiMADS04*	Seita.1G148200.1	1	PF00319	441	146	4.76	16.05	1	M
*SiMADS05*	Seita.1G183300.1	1	PF00319	777	258	7.72	27.48	0	M
*SiMADS06*	Seita.1G209300.1	1	PF00319; PF01486	723	240	8.85	27.45	7	AGL17
*SiMADS07*	Seita.1G272300.1	1	PF00319	693	230	8.68	24.24	0	M
*SiMADS08*	Seita.1G273400.1	1	PF00319; PF01486	762	253	8.85	28.81	7	AGL6
*SiMADS09*	Seita.1G308200.1	1	PF00319; PF01486	729	242	9.27	27.64	7	AGL17
*SiMADS10*	Seita.1G328500.1	1	PF00319; PF01486	687	228	5.41	25.44	7	SVP
*SiMADS11*	Seita.2G002300.1	2	PF00319; PF01486	810	269	9.31	30.85	7	AP1(SQUA)
*SiMADS12*	Seita.2G026600.1	2	PF00319	1182	393	5.76	43.49	1	M
*SiMADS13*	Seita.2G086800.1	2	PF00319	741	246	5.64	26.81	0	M
*SiMADS14*	Seita.2G115700.1	2	PF00319	333	110	4.79	11.96	0	M
*SiMADS15*	Seita.2G266600.1	2	PF00319; PF01486	729	242	9.17	27.79	7	SEP
*SiMADS16*	Seita.2G383000.1	2	PF00319; PF01486	759	252	9.11	28.53	7	AP1(SQUA)
*SiMADS17*	Seita.3G055200.1	3	PF00319	1434	477	4.41	51.33	1	M
*SiMADS18*	Seita.3G073000.1	3	PF00319; PF01486	867	288	9.23	32.59	7	AG
*SiMADS19*	Seita.3G098400.1	3	PF00319; PF01486	795	264	9.1	29.85	6	AG
*SiMADS20*	Seita.3G098800.1	3	PF00319; PF01486	648	215	9.34	24.66	7	AGL12
*SiMADS21*	Seita.3G236800.1	3	PF00319; PF01486	648	215	8.45	25.11	6	PI(GLO)
*SiMADS22*	Seita.3G278000.1	3	PF00319	294	97	4.45	10.85	1	M
*SiMADS23*	Seita.3G280400.1	3	PF00319	588	195	10.25	21.22	0	M
*SiMADS24*	Seita.3G301600.1	3	PF00319	471	156	5.44	17.25	0	M
*SiMADS25*	Seita.3G358100.1	3	PF00319; PF01486	735	244	7.07	27.42	7	AP1(SQUA)
*SiMADS26*	Seita.4G062600.1	4	PF00319; PF01486	684	227	7.59	26.22	7	SEP
*SiMADS27*	Seita.4G077200.1	4	PF00319; PF01486	669	222	5.36	24.4	6	SVP
*SiMADS28*	Seita.4G093200.1	4	PF00319	1191	396	4.99	43.22	9	MIKC*
*SiMADS29*	Seita.4G160200.1	4	PF00319	699	232	10.27	25.94	2	M
*SiMADS30*	Seita.4G163500.1	4	PF00319	645	214	9.59	23.2	0	M
*SiMADS31*	Seita.4G177800.1	4	PF00319	552	183	7.91	20.08	0	M
*SiMADS32*	Seita.4G184600.1	4	PF00319	510	169	5.44	18.55	0	M
*SiMADS33*	Seita.4G219100.1	4	PF00319	468	155	5.11	17.11	1	M
*SiMADS34*	Seita.4G238000.1	4	PF00319	960	319	5.62	34.27	0	M
*SiMADS35*	Seita.4G268200.1	4	PF00319; PF01486	717	238	4.97	26.54	4	Bsisiter
*SiMADS36*	Seita.4G277600.1	4	PF00319; PF01486	690	229	8.74	26.11	6	AP3(DEF)
*SiMADS37*	Seita.5G033100.1	5	PF00319	753	250	9.61	27.8	0	M
*SiMADS38*	Seita.5G036500.1	5	PF00319; PF01486	690	229	7.73	25.99	6	AGL17
*SiMADS39*	Seita.5G101300.1	5	PF00319; PF01486	507	168	8.9	18.66	4	FLC
*SiMADS40*	Seita.5G114500.1	5	PF00319	750	249	8.59	27.84	0	M
*SiMADS41*	Seita.5G143100.1	5	PF00319; PF01486	774	257	9.07	29.07	7	AG
*SiMADS42*	Seita.5G160200.1	5	PF00319	1347	448	5.25	46.65	0	M
*SiMADS43*	Seita.5G220600.1	5	PF00319	552	183	5.03	20.8	1	M
*SiMADS44*	Seita.5G225300.1	5	PF00319	1260	419	5.89	46.71	0	M
*SiMADS45*	Seita.5G303200.1	5	PF00319; PF01486	591	196	6.55	22.68	4	OsMADS32-like
*SiMADS46*	Seita.5G404600.1	5	PF00319; PF01486	630	209	7.1	24.21	6	PI(GLO)
*SiMADS47*	Seita.5G406700.1	5	PF00319; PF01486	810	269	9.11	29.89	6	AG
*SiMADS48*	Seita.5G424200.1	5	PF00319	984	327	5.18	34.94	0	M
*SiMADS49*	Seita.5G425300.1	5	PF00319	1389	462	5.11	49.4	0	M
*SiMADS50*	Seita.5G432700.1	5	PF00319	840	279	10.58	29.46	4	FLC
*SiMADS51*	Seita.6G013400.1	6	PF00319; PF01486	678	225	6.61	25.39	6	AGL12
*SiMADS52*	Seita.6G156800.1	6	PF00319; PF01486	702	233	8.89	26.76	6	AGL17
*SiMADS53*	Seita.6G194800.1	6	PF00319	1035	344	4.58	37.43	11	MIKC*
*SiMADS54*	Seita.6G223400.1	6	PF00319; PF01486	738	245	8.75	28.42	7	SEP
*SiMADS55*	Seita.6G223600.1	6	PF00319	528	175	8.97	19.88	4	FLC
*SiMADS56*	Seita.6G223700.1	6	PF00319	372	123	10.13	14.22	1	FLC
*SiMADS57*	Seita.7G040500.1	7	PF00319	456	151	4.49	16.64	1	M
*SiMADS58*	Seita.7G109700.1	7	PF00319; PF01486	609	202	7.06	23.29	6	AGL12
*SiMADS59*	Seita.7G110000.1	7	PF00319	330	109	9.94	12.49	2	AGL12
*SiMADS60*	Seita.7G125400.1	7	PF00319; PF01486	717	238	7.72	27.18	7	AGL17
*SiMADS61*	Seita.7G210200.1	7	PF00319; PF01486	765	254	8.59	28.48	7	AGL6
*SiMADS62*	Seita.7G235900.1	7	PF00319; PF01486	738	245	9.27	28.01	5	Bsisiter
*SiMADS63*	Seita.8G182900.1	8	PF00319	627	208	6.21	22.05	0	M
*SiMADS64*	Seita.8G220800.1	8	PF00319	1110	369	6.2	40.94	10	MIKC*
*SiMADS65*	Seita.9G088700.1	9	PF00319; PF01486	741	246	6.45	27.88	7	SEP
*SiMADS66*	Seita.9G088900.1	9	PF00319; PF01486	750	249	9.33	28.47	7	AP1(SQUA)
*SiMADS67*	Seita.9G237300.1	9	PF00319	1098	365	5.03	40.09	4	M
*SiMADS68*	Seita.9G270800.1	9	PF00319	1230	409	5.18	43.79	1	M
*SiMADS69*	Seita.9G342700.1	9	PF00319; PF01486	678	225	9.6	25.62	6	SOC1(TM3)
*SiMADS70*	Seita.9G393900.1	9	PF00319	717	238	5.6	26.57	0	M
*SiMADS71*	Seita.9G513900.1	9	PF00319; PF01486	687	228	8.47	25.63	7	SVP
*SiMADS72*	Seita.9G561000.1	9	PF00319	186	61	10.65	6.97	0	SOC1(TM3)

The MADS-box protein sequences were aligned using ClustalX 2.0 with the default parameters (Larkin et al., 2007). A phylogenetic tree was constructed using the maximum likelihood method with 1,000 bootstrap replicates as implemented in MEGA 7 (Sudhir et al., 2016).

### Chromosomal Localization and Gene Duplication Analysis of *MADS-Box* Genes in Foxtail Millet

All identified *MADS-box* genes were mapped to the nine foxtail millet chromosomes using the information obtained from the foxtail millet database using MapDraw software (Liu and Meng, 2003). Gene duplication analysis of foxtail millet *MADS-box* genes was conducted using the screening criteria proposed by Gu et al. (2002). The software KaKs_calculator (Zhang et al., 2006) was used to calculate the Ka/Ks values.

### Predicted Protein Motifs and Gene Structure Characterization of Foxtail Millet *MADS-Box* Genes

The Multiple EM for Motif Elicitation (MEME) online tool^5^ was used to discover motifs in the predicted foxtail millet MADS-box proteins (Bailey et al., 2015). A MEME search was executed with the following parameters: motif count is 20; motif width ranges from 6 to 200 (inclusive) amino acids, and any number of repetitions (Arora et al., 2007). The detailed amino acid sequences of the 20 motifs are shown in Supplementary Table 2.

The coding sequences (CDS) and genomic DNA (gDNA) sequences of foxtail millet *MADS-box* genes were downloaded from the Phytozome database in order to predict gene structures. The Gene Structure Display Server 2.0 (GSDS 2.0^6^) online website was used to display the structures of the *MADS-box* genes (Hu et al., 2014).

### *Cis*-Regulatory Element Analysis and Expression Analysis of Foxtail Millet *MADS-Box* Genes

In general, the 2,000 bp sequence located upstream of the initiation codon of a gene is considered to be the promoter region (Zhao et al., 2016). Therefore, the promoter sequences of all foxtail millet *MADS-box* genes were acquired from the Phytozome website. The primary *cis*-regulatory elements (CREs) in the promoter regions were then predicted using the online tool New PLACE^7^ (Higo et al., 1999). All predicted CREs obtained were counted and classified (Supplementary Table 3). The distribution of *cis*-elements related to abiotic stresses in the promoter regions were shown in Table 3.

The RNA-seq data for foxtail millet plants under drought stress was extracted from our previous research to investigate the expression of *SiMADS*-box genes in response to drought (Yu et al., 2018). The transcriptome sequence data for different tissues including the leaves, roots, stems, and tassel inflorescences were downloaded from Expression Atlas^8^ (Zhang G. Y. et al., 2012). Using the FPKM values (fragments per kilobase of transcript per million mapped reads) we obtained, a heat map of *SiMADS-box* gene expression in the different tissues was drawn with EvolView (Zhang H. K. et al., 2012).

### Plant Materials, Growth Conditions, and Treatments

In this study, wild type *Arabidopsis* (ecotype Col-0) and rice (cv. ‘Nipponbare’) were used as recipients in the transformation experiments. Foxtail millet variety ‘Yugu1’ was used for the cDNA amplification, promoter sequence amplification, and expression analysis of the *SiMADS51* gene. The seeds of foxtail millet were sown in nutrition soil and grown at 28°C in a greenhouse at ∼65% relative humidity and a 14 h/10 h (day/night) photoperiod. Three-week-old seedlings were treated with various abiotic stresses and exogenous hormones, including drought (15% PEG-6000 to simulate drought conditions), NaCl (100 mM), ABA (100 μM), and GA (100 μM). Leaves of foxtail millet seedlings were sampled at 0, 1, 3, 6, 12, and 24 h after the treatments and frozen immediately in liquid nitrogen for RNA extraction.

### RNA Isolation, RT-PCR, and qPCR Analysis

A Plant Total RNA Kit (ZP405, Beijing Zoman Biotechnology Co., Ltd.) was used for total RNA isolation following the kit instructions. For cDNA synthesis we used the EasyScript^®^ One-Step gDNA Removal and cDNA Synthesis SuperMix kit (AE311-02, Transgen, Beijing). TransStart^®^ Top Green qPCR SuperMix (+Dye II) kit (AQ132-21, Transgen, Beijing) was used for qPCR assays to determine the relative level of gene expression. The foxtail millet *actin* gene (GenBank ID: AF288226), *Arabidopsis actin2* gene (*At3g18780*), and rice *actin* gene (*LOC_Os03g50885.1*) were used as internal controls for normalization of gene expression in the three species. The relevant gene-specific primers used in this study are given in Supplementary Table 6. The experimental data was analyzed using the 2^–ΔΔ*Ct*^ method of Livak and Schmittgen (2001).

### Subcellular Localization

To investigate the subcellular localization, the coding sequences excluding the termination codons of 10 *SiMADS-box* genes were cloned into the plant expression vector p16318h-GFP. The control plasmid and the recombinant plasmids were introduced into rice protoplasts separately as described previously (Zhang et al., 2011). The transfected protoplasts were incubated for 18 h in darkness at 22°C, after which they were examined using a confocal laser scanning microscope.

### Generation of Transgenic *Arabidopsis* and Rice Plants Overexpressing the *SiMADS51* Gene

To obtain transgenic plants, the full-length coding region of *SiMADS51* was amplified from cDNA prepared from foxtail millet RNA (leaves sampled at 0 h). The coding sequence of *SiMADS51* excluding the termination codon was amplified and cloned into the plant expression vectors pCAMBIA1302 and pCAMBIA1305. To generate transgenic *Arabidopsis* plants, the fusion plasmid *pCAMBIA1302:SiMADS51* was transfected into *Agrobacterium tumefaciens* strain GV3101 which was then used to transform *Arabidopsis* Col-0 plants by floral dipping method (Clough and Bent, 1998). 0.5X MS solid medium (2.5 g/L phytagel) containing 30 mg/L hygromycin was used to screen transgenic *Arabidopsis* lines (Zhao et al., 2017). Transgenic *Arabidopsis* seedlings were cultured at 22°C with ∼65% relative humidity and a 16 h light/8 h dark photoperiod in a climate-controlled chamber. To obtain transgenic rice plants, the fusion plasmid *pCAMBIA1305:SiMADS51* was transformed into *Agrobacterium tumefaciens* strain EHA105, and the recombinant strain was used to transfect rice embryonic calli according to the procedure detailed in Sallaud et al. (2003). Transgenic rice plants were selected by hygromycin (50 mg/L) and planted in soil substrate in a containment greenhouse under daily cycle of 28°C 14 h light/22°C 10 h dark (Khong et al., 2015). All constructs were sequence-verified before they were used for plant transformation. *SiMADS51*-positive transgenic plants were screened using PCR and cultured to the T_3_ generation. The expression levels of *SiMADS51* in the T_3_-generation transgenic lines were determined by RT-qPCR, and the lines with the highest expression levels were then used for the further evaluation of drought resistance.

### Drought-Resistance Assessment of the Transgenic Plants

For the seed germination test, seeds of the wild type (WT; Col-0) and three *SiMADS51*-overexpressing (OE) *Arabidopsis* lines were sterilized and sown on 0.5X MS medium with or without 6% PEG-6000 or 9% PEG-6000. All the seeds were vernalized at 4°C for 3 days in the dark before being transferred to a climate-controlled chamber at 22°C with ∼65% relative humidity and a 16 h light/8 h dark photoperiod. The numbers of germinated seeds were recorded every 12 h.

For the root growth test, 8-day-old WT and OE *Arabidopsis* seedlings grown on 0.5X MS medium were transferred to 0.5X MS medium with or without 9% PEG-6000. The transferred seedlings were cultured in the climate chamber for a week. The total root lengths were then measured using a root system scanner.

To evaluate drought-resistance in adult-stage transgenic *Arabidopsis* plants, 8-day-old WT and OE *Arabidopsis* seedlings grown on 0.5X MS medium were transferred to soil with suitable moisture. Water was withheld from 2-week-old soil-grown *Arabidopsis* seedlings until they wilted (about 2 weeks). The plants were then re-watered and allowed to recover for 1 week in the growth chamber.

To assay drought stress in transgenic rice plants, hydroponics experiment under PEG-6000 simulated drought conditions were conducted in the growth chamber. Rice seeds were sterilized with 2.5% sodium hypochlorite (NaClO) for 30 min and germinated in tap water at 28-degree incubator for 2 days. Germinated seeds were cultured in 0.5X MS liquid medium for 2 weeks under the conditions described above. The seedlings were then transferred to 0.5X MS liquid medium containing 15% or 20% PEG-6000 and continued to grow until the seedlings were all wilted (about 1 week). Next, the seedlings were transferred to 0.5X MS liquid medium and allowed to recover for 1 week. All liquid media were changed every 2 days.

### Physiological Measurements

For physiological measurements, the leaf samples from the control and transgenic plants were collected before and after stress treatments. The measurements of proline (Pro), chlorophyll, and MDA contents and the assays of SOD and POD activity were conducted using the corresponding test kits (Comin, China) following the manufacturer’s protocols. A Varioskan LUX Multimode Microplate Reader (Thermo Fisher Scientific, United States) was used to measure the absorbance values. All measurements were repeated three times with three biological replicates.

### Data Analysis

All experiments above were replicated three times independently. GraphPad Prism 5.0 software was used for statistical analyses. Statistical analysis of the data was performed with Student’s *t*-test and ANOVA (a one-way analysis of variance). The significant or extremely significant differences (*p* < 0.05, *p* < 0.01) between two sets data are marked with single (^∗^) or double (^∗∗^) asterisks, respectively, in the figures.

## Results

### Identification, Characterization, and Phylogenetic Analysis of Foxtail Millet *MADS-Box* Genes

A total of 72 candidate genes encoding MADS-box domain (SRF-TF) were identified in the foxtail millet (*Setaria italica* L.) genome and named as *SiMADS01*∼*SiMADS72* based on their chromosomal locations. The predicted open reading frames (ORFs) of the *SiMADS-box* genes ranged from 186 to 1,434 bp, and the encoded amino acid sequences varied from 61 to 477 aa. The predicted molecular weights (MW) of the SiMADS-box proteins ranged from 6.79 to 53.18 kDa, with predicted isoelectric points (p*I*) that ranged from 4.41 to 10.65 (Table 1).

**FIGURE 1:**
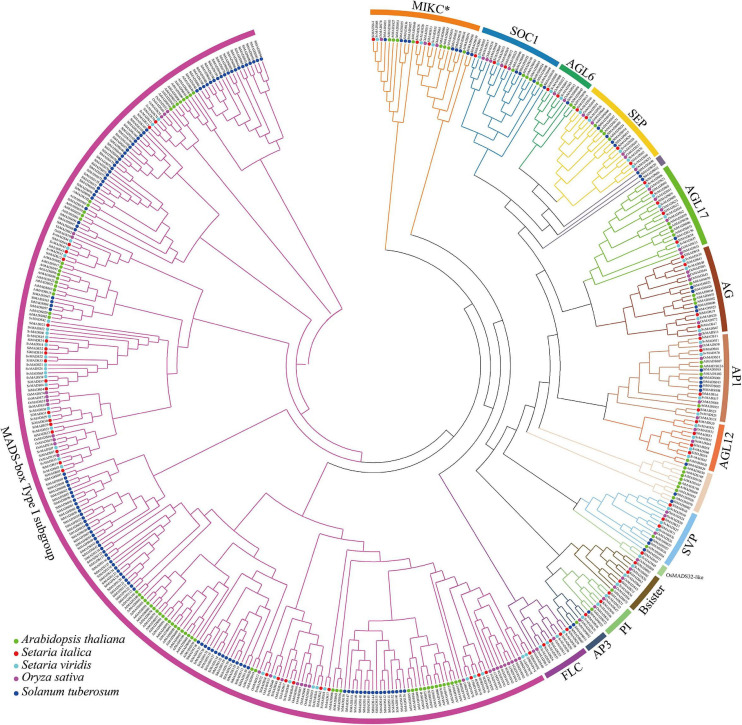
Phylogenetic analysis of MADS-box transcription factor genes in foxtail millet, *Arabidopsis*, *Setaria viridis*, potato (*Solanum tuberosum*), and rice (*Oryza sativa* L.). A total of 488 MADS-box protein sequences were used to construct the ML (maximum likelihood) tree. The different clades were marked respectively in different colors.

**FIGURE 2:**
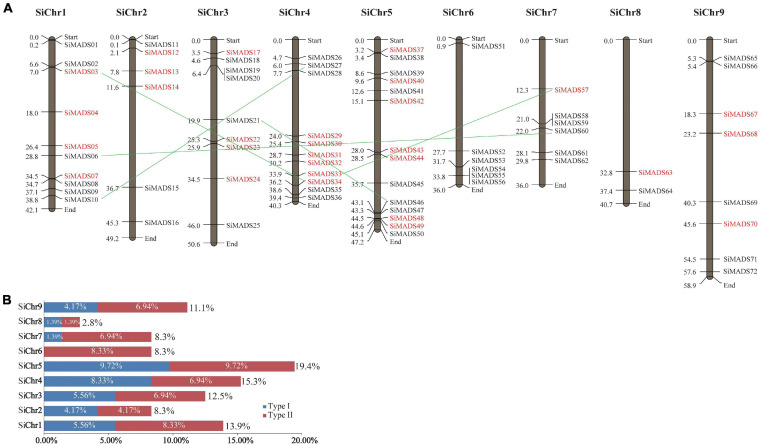
Chromosomal location of foxtail millet *MADS-box* genes. **(A)** The 72 *SiMADS-box* genes distributed on the nine foxtail millet chromosome. Type I and type II genes are colored red and black, respectively. The duplicated genes are connected by green lines between the two relevant chromosomes. **(B)** The percentages of *SiMADS-box* genes on each chromosome.

To investigate the phylogenetic relationships among MADS-box proteins in monocotyledons and dicotyledons, a phylogenetic tree was constructed with 488 MADS-box protein sequences from *Arabidopsis* (109), potato (*Solanum tuberosum*) (156), rice (*Oryza sativa* L.) (75), foxtail millet (72), and *Setaria viridis* (76). The details of each gene are shown in Supplementary Table 1. We also constructed a second phylogenetic tree with only the 72 foxtail millet MADS-box proteins. Based on amino acid sequence similarities and the previous classification of MADS-box proteins from *Arabidopsis* and rice (Paøenicová et al., 2003; Arora et al., 2007), the 72 *MADS-box* genes in the foxtail millet genome were divided into two major phylogenetic clades: 29 Type I (M-type) and 43 Type II (MIKC-type) (Figures 1, 2A). The 43 Type II *MADS-box* genes were then further divided into the 14 major subclades: SEP (4 proteins), AGL6 (2), AP1/SQUA (4), FLC (4), SOC1/TM3 (3), SVP/StMADS11 (3), PI/GLO (2), AP3/DEF (1), AG/STK (4), AGL17 (5), AGL12 (4), Bsister/GGM13 (3), OsMADS32-like (1), and MIKC^∗^ (3) (Figures 1, 2A). As can be seen from Figure 1, most of the type I *MADS-box* genes from each species clustered into one clade, showing a sister-group relationship. All of the foxtail millet Type II proteins clustered with their counterparts from *Arabidopsis*, potato, *Setaria viridis*, and rice with high bootstrap support apart from *SiMADS45*, which has no known ortholog in *Arabidopsis* and potato. In particular, the AGL17, AGL12, Bsister, and PI (GLO) subclades are significantly expanded in foxtail millet, rice, and *Setaria viridis*, compared with *Arabidopsis* and potato (Figure 1).

### Chromosomal Locations and Gene Duplication Analysis of Foxtail Millet *MADS-Box* Genes

The physical positions of the *MADS-box* genes on the foxtail millet chromosomes were visualized with Mapdraw software. As shown in Figure 2A, the 72 *MADS-box* genes are distributed on nine foxtail millet chromosomes. Chromosome 8 contains the fewest *MADS-box* genes (∼2.8%), while chromosomes 5, 4, and 1 contain the most (19.4%, 15.3%, and 13.9%, respectively), and account for nearly half of all the *MADS-box* genes (Figure 2B). Also, we found that the Type I and Type II *MADS-box* genes showed an uneven distribution on the foxtail millet chromosomes. There were no Type I genes identified on chromosome 6, whereas Type II genes are distributed across all nine chromosomes (Figures 2A,B). The numbers of Type I and Type II genes on chromosomes 2, 5, and 8 are equal, while there are more Type II genes than Type I genes on chromosomes 1, 3, 7, and 9 (Figure 2B). Also, some *MADS-box* genes from the same subclades tend to cluster together in one region of the chromosome. For example, *SiMADS55* and *SiMADS56*, and *SiMADS58* and *SiMADS59*, which belong to the FLC and AGL12 subclades, respectively, are tightly linked on chromosomes 6 (*SiMADS55/56*) and 7 (*SiMADS58/59*) (Figure 2A).

For gene duplication analysis, the coding sequences of the 72 foxtail millet *MADS-box* genes were used as queries in BLAST searches against all other *SiMADS-box* genes using an *E*-value < 1e^–10^ and identity >85%. After the screening, only five pairs (*SiMADS03* and *SiMADS34*, *SiMADS06* and *SiMADS60*, *SiMADS10* and *SiMADS27*, *SiMADS21* and *SiMADS46*, and *SiMADS33* and *SiMADS57*) met the search criteria, and both members of each pair are located on different chromosomes (Figure 2A and Table 2). These results mean that the duplicated *MADS-box* genes are likely to have been generated by segmental duplication, which would produce many homologs on different chromosomes (Duan et al., 2015). The Ka, Ks, and Ka/Ks values of the homologs were calculated using a KaKs_calculator and are shown in Table 2. Based on the Ka/Ks ratios, we determined that the evolution of foxtail millet *MADS-box* genes was mainly accelerated by purifying selection (Hurst, 2002).

**TABLE 2:** The Ka, Ks, and Ka/Ks values of the duplicated *SiMADS-box* genes.

Gene name	Ka	Ks	Ka/Ks	*P*-Value
*SiMADS03* and *SiMADS34*	1.13128	0.755894	1.49661	1.30E-12
*SiMADS06* and *SiMADS60*	0.130958	0.886409	0.14774	7.15E-41
*SiMADS10* and *SiMADS27*	0.810601	1.68021	0.48244	1.46E-24
*SiMADS21* and *SiMADS46*	0.0855293	1.82945	0.0467514	0
*SiMADS33* and *SiMADS57*	0.183993	0.336445	0.546873	0.00704695

**TABLE 3:** Number of *cis*-elements in the promoter region of 10 *SiMADS-box* genes.

Element Name	*SiMADS01*	*SiMADS10*	*SiMADS25*	*SiMADS28*	*SiMADS36*	*SiMADS45*	*SiMADS51*	*SiMADS55*	*SiMADS56*	*SiMADS58*
ABREs	3	5	4	3	7	4	3	3	3	
MYB	4	3	3	6	9	7	6	3	2	14
MYC	10	2	18	7	7	10	4	9	9	17
W BOX	5	2	3	6	8	3	8	7	7	6
LTRE	1	2		3		1		2	1	1
BIHD1OS	3	1	1	4	2	1		3	1	3
CCAATBOX1	3	1	1	2		3	1	3	1	2
ACGTATERD1	5	1	4	2	5	7	4	2	7	
ELRECOREPCRP1	1						1		1	1
CBFHV	1	1	2	4		1		1		
GAREs	1				1	1				
GCCCORE			3	2	1	2			2	
DRECRTCOREAT		1		2				1		
Total	37	21	39	41	40	40	27	34	34	44

### Conserved Protein Motifs and Structure of Foxtail Millet MADS-Box Protein Genes

**FIGURE 3:**
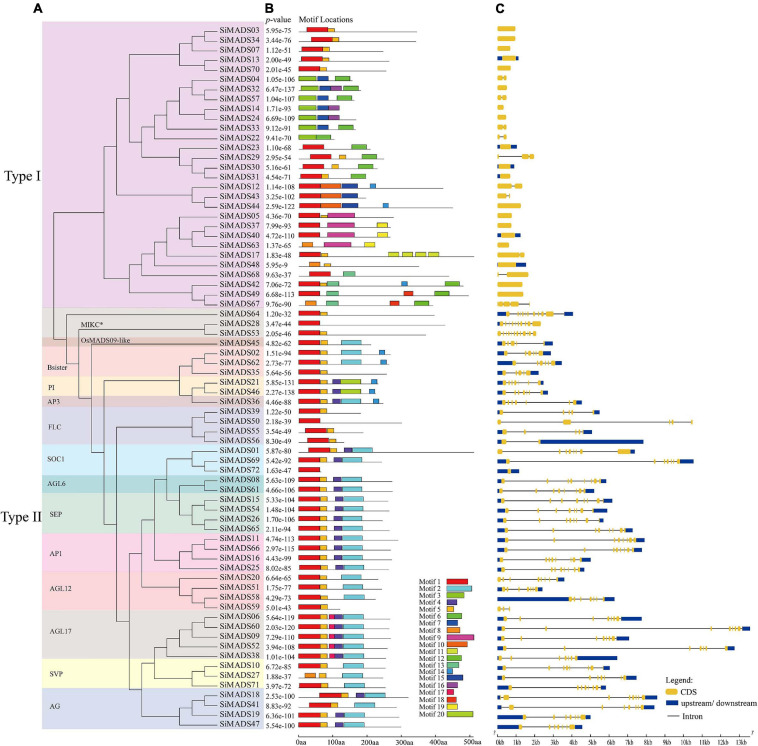
Evolutionary tree, conserved motif compositions, and gene structures of foxtail millet *MADS-box* genes. **(A)** A total of 72 foxtail millet MADS-box protein sequences were used to construct the ML (maximum likelihood) tree. The different clades were framed by rectangles filled with different colors. **(B)** Motif locations of the 72 foxtail millet MADS-box proteins. Different motifs are represented by different colored boxes. Motifs 1, 3, and 8 represent the SRF-TF (MADS) domain, motifs 2 and 20 represent the K-box domain, motifs 4, 5, and 17 represent the intervening (I) region. The box length represents motif length. Corresponding p-value are shown on the left of panel **(B)**. **(C)** The exon-intron structure analyses of 72 foxtail millet *MADS-box* genes. The lengths of the exons and introns of each *MADS-box* genes are displayed proportionally. The yellow boxes represent exons, the black lines represent introns, and the blue boxes represent 5′ and 3′ non-coding regions.

The MEME on line tool (see text footnote 5) was used to identify the conserved motifs in the 72 predicted foxtail millet MADS-box proteins. A total of 20 conserved motifs (motif 1 to motif 20), were identified (Figure 3B). Detailed motif information is given in Supplementary Table 2. As can be seen in Figure 3B, Type I and Type II MADS-box proteins contain 15 and eight main motifs, respectively. The Type I MADS-box proteins exhibit more motif variation, and this is likely due to their non-conserved C-terminal regions. As anticipated, some particular motifs are specific to each family; for example, motifs 3, 9, 10, and 11 are found in Type I proteins, and motifs 2, 19, and 20 are found in Type II proteins (Figures 3A,B). The specificity of the protein motifs probably accounts for the functional diversity of the Type I and Type II family proteins. In general, the MADS-box proteins with similar motifs tended to cluster together in the phylogenetic analysis [the PI (GLO), AP1, and AGL6 subclades, for example], which implies that members of the same subclade have similar functions (Parenicová et al., 2003). However, we also found that some members, including SiMADS27, SiMADS35, and SiMADS59, differed from other members of the same subclade in terms of the particular motifs (Figures 3A,B). These differences might be due to the evolution of the MADS-box protein genes in foxtail millet.

To investigate the gene structures, the intron-exon organization of the foxtail millet *MADS-box* genes were analyzed using the GSDS online tool (see text footnote 6). As shown in Figures 3A,C, we found that the number of introns in foxtail millet *MADS-box* genes varied from 0 to 11, and the number of introns in Type I and Type II protein genes showed obvious differences. To be specific, 27 of 29 (93.1%) Type I genes contained either none or one intron, except for *SiMADS29* and *SiMADS67*, while 40 of 43 (93%) Type II genes had at least four introns, although *SiMADS56*, *SiMADS59*, and *SiMADS72* were among the exceptions (Figures 3A,C). This distribution of introns is analogous to that reported in *Arabidopsis* and rice (Parenicová et al., 2003; Arora et al., 2007). The PI (GLO), AP1 and AGL6 subclade proteins not only possess similar conserved motifs, but they also have similar numbers of introns, with the only differences between the members being the lengths of the introns and exons (Figures 3A–C). Also, within the same subclade, the proteins in which the motifs differ greatly from the others (e.g., SiMADS27, SiMADS35, and SiMADS59), the genes also differ greatly in the arrangement of the introns and exons (Figures 3A–C).

### Promoter *Cis*-Regulatory Element Enrichment Analysis of Foxtail Millet *MADS-Box* Genes

**FIGURE 4:**
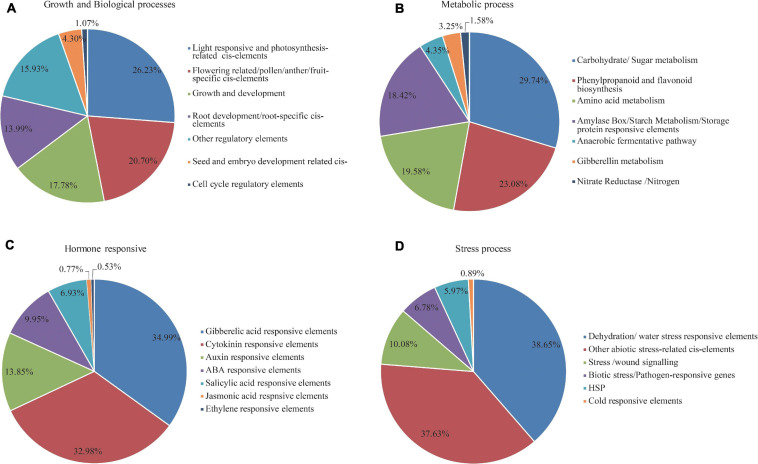
Percentage distribution of cis-regulator elements (CREs) in the promoters of *SiMADS-box* genes based upon the putative functions. **(A)** Growth and biological process responsive elements. **(B)** Metabolism responsive elements. **(C)** Stress responsive elements. **(D)** Hormone responsive elements.

Regulation of gene expression is mainly dependent on the presence of CREs in the promoter region. To investigate the potential CREs present in the *MADS-box* gene family in foxtail millet, the 2000 bp of genomic DNA sequence upstream of the coding region of each gene was retrieved from the Phytozome website and searched against the New PLACE database. This analysis predicted 13,915 putative CREs in the 72 *SiMADS*-box genes (Supplementary Table 3). All the CREs can be divided into four broad categories based on their functions and response to stimuli (Supplementary Table 3 and Figure 4). Statistical analysis showed that growth and biological process responsive elements comprised 48.95% of all CREs, followed by metabolism responsive elements (22.79%), and stress responsive elements (16.12%). Hormone responsive elements represented the lowest proportion (12.14%) of the CREs (Supplementary Table 3). For the growth and biological process responsive elements, light- and photosynthesis-related cis-elements accounted for 26.23% of the total (Figure 4A). Amongst the metabolism-related elements, the proportion of carbohydrate/sugar metabolism responsive elements (29.74%) followed by phenylpropanoid and flavonoid biosynthesis-related *cis*-elements (23.08%) was highest (Figure 4B). As shown in Figures 4C, numerous GA, Auxin, ABA, and SA response elements were identified upstream of the *SiMADS*-box genes, among which the GA response elements were the most abundant. Additionally, a large number of stress responsive *cis*-elements, which are involved in dehydration/water stress, wound signaling, and defense responsiveness, were also found in the *SiMADS*-box gene promoters (Figure 4D). These results imply the possible involvement of the *SiMADS*-box genes in plant development and stress tolerance.

In our previous study, was performed RNA-seq analysis of drought-treated foxtail millet seedlings (Yu et al., 2018). Twenty-five *SiMADS*-box genes that showed a transcriptional response to drought stress were identified; among them, the relative expression levels of 10 *SiMADS*-box genes were found to be up-regulated in response to drought stress, and the differences were statistically significant (*P* < 0.05) (Supplementary Table 4). Interestingly, all of these 10 *SiMADS*-box genes belong to different Type II MADS-box subclades. Moreover, each of the 10 *SiMADS*-box genes contained at least 21 stress- or hormone-related CREs in their promoter regions (Table 3). This demonstrates that a majority of Type II *MADS-box* genes in foxtail millet may be involved in the drought stress response.

### Tissue-Specific Expression of *SiMADS-Box* Genes and Subcellular Localization of SiMADS-Box Proteins

**FIGURE 5:**
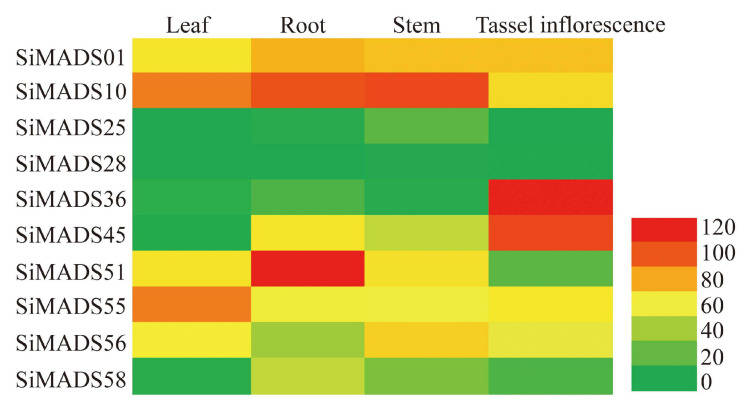
The transcript accumulation of ten *SiMADS-box* genes in various foxtail millet tissues (root, leaf, stem, and tassel inflorescence). The transcriptome data of foxtail millet for different tissues were obtained from Expression Atlas. A heatmap was generated using EvolView. Transcript levels are indicated by different colors.

To further analyze the expression patterns of the 10 Type II *SiMADS*-box genes in various tissues and organs, we downloaded the transcriptome sequencing data for different tissues of foxtail millet. The expression profiles of 10 *SiMADS*-box genes in leaf, root, stem and tassel inflorescence are given in Supplementary Table 5 and shown in a heatmap in Figure 5. The results show that the 10 *SiMADS*-box genes exhibited large differences in their expression profiles. For example, *SiMADS36*, *SiMADS51*, and *SiMADS55* showed their highest transcript levels in the tassel inflorescence, roots, and leaves, respectively, and were expressed at lower levels in other tissues; *SiMADS01* and *SiMADS10* were expressed at moderate to high levels in the leaves, roots, stems, and tassel inflorescence, while *SiMADS25* and *SiMADS28* were weakly expressed in all tissues and organs examined (Figure 5).

Subcellular localization information is important in the study of protein function. To investigate the subcellular distribution of the predicted SiMADS-box proteins, the coding sequences without the stop codons of the *SiMADS*-box genes were fused in frame with the gene for green fluorescent protein (GFP), and the constructs were transformed into rice protoplasts. We observed that the GFP fluorescence signals for the 10 SiMADS-box protein fusions were only found in the nucleus (Supplementary Figure 1), suggesting that these SiMADS-box proteins function mainly in the nucleus.

### *SiMADS-Box* Gene Expression Is Induced by Multiple Abiotic Stresses

**FIGURE 6:**
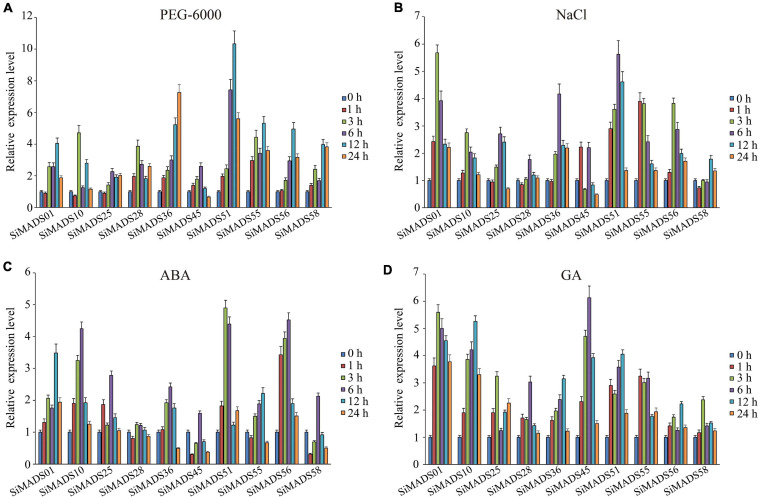
Expression level of ten foxtail millet *MADS-box* genes under **(A)** PEG-6000, **(B)** NaCl, **(C)** ABA, and **(D)** GA treatment. The data were normalized to the foxtail millet *actin* gene (GenBank ID: AF288226). Three biological replicates were performed, and the values are presented as the means ± SD.

Previously published studies have suggested that *MADS-box* genes from different species are involved in the regulation of abiotic stresses and hormone response processes (Zhang G. Y. et al., 2012; Khong et al., 2015; Castelán-Muñoz et al., 2019). To further investigate the possible involvement of the 10 *SiMADS*-box genes that are upregulated by drought in the response to abiotic stresses, we measured their expression patterns in response to PEG-6000, NaCl, ABA, and GA by RT-qPCR (Figure 6). The quantitative analysis results indicated that the 10 *SiMADS-box* genes had distinctly different transcriptional responses to the various abiotic stress or phytohormone treatments. Under the osmotic stress induced by PEG-6000, the transcription levels of *SiMADS51*, *SiMADS36*, and *SiMADS55* were dramatically up-regulated (>5-fold) and reached a peak at 12 h, 24 h, and 12 h, respectively (Figure 6A). In the salt stress treatment, the expression levels of *SiMADS01* and *SiMADS51* were up-regulated five-fold compared to the controls, and the expression of the two genes peaked at 6 h and 12 h, respectively (Figure 6B). Following ABA stress treatment, the expression of *SiMADS51*, *SiMADS56*, and *SiMADS10* was up-regulated four-fold, and the highest transcription levels occurred at 3 h, 6 h, and 6 h, respectively, while expression of the *SiMADS28* gene increased only slightly in response to ABA (<2-fold) (Figure 6C). When the seedlings were treated with GA, the transcript levels of the 10 *SiMADS-box* genes were at least twice that of the control; *SiMADS45* showed the highest overall expression level, which occurred at 6 h (Figure 6D). These results suggest that foxtail millet MADS-box TFs may play diverse roles in plant responses to abiotic stresses.

### Overexpression of *SiMADS51* Affects Seed Germination in Response to PEG-6000 Treatment in *Arabidopsis*

**FIGURE 7:**
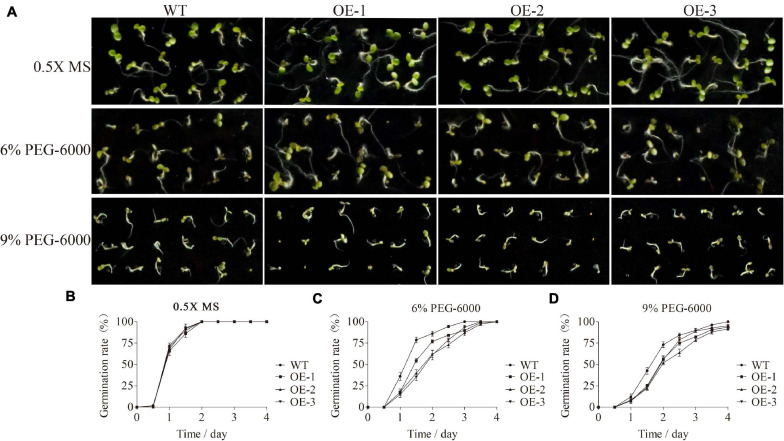
Germination assays of wild-type (WT) and *SiMADS51* transgenic *Arabidopsis* seeds under PEG-6000 treatments. **(A)** Phenotypes of WT and *SiMADS51* transgenic *Arabidopsis* seeds on 0.5X Murashige and Skoog (MS) medium with or without 6% PEG-6000 and 9% PEG-6000, respectively. **(B–D)** Germination rates of WT and *SiMADS51* transgenic *Arabidopsis* seeds on 0.5X MS medium, 0.5X MS medium containing 6% PEG-6000, and 0.5X MS medium containing 9% PEG-6000 at different time points. Date for each time point are means of three biological replicates.

Based on the gene expression analysis, *SiMADS51*, which belongs to the AGL12 subclade and is induced strongly by PEG-6000, was selected for further stress tolerance assays in transgenic *Arabidopsis* plants. The relative expression levels of *SiMADS51* gene in different line plants were shown in Supplementary Figure 2A. Homozygous T_3_-generation transgenic *Arabidopsis* seeds were used in the germination test. After surface sterilization, the *Arabidopsis* seeds were sown on 0.5X MS medium with or without 6% PEG-6000 and 9% PEG-6000. Statistical analyses of the results showed that there were no significant differences in germination between wild-type (WT) and transgenic *Arabidopsis* lines (OE-1, OE-2, and OE-3) on 0.5X MS medium (Figures 7A,B), indicating that overexpression of *SiMADS5*1 probably has no effect on plant growth and development under normal conditions. In the presence of 6% PEG-6000 and 9% PEG-6000, germination of both wild-type and transgenic lines was inhibited; nonetheless, *SiMADS51*-overexpressing lines had lower germination percentages than WT (Figures 7A,C,D). For example, on 0.5X MS medium supplemented with 9% PEG-6000, the germination rates of seeds from each transgenic line within 2 days was 56.5% (OE-1), 51.9% (OE-2), and 55.6% (OE-3), significantly lower than the 73.1% germination rate in the wild-type seeds.

### Overexpression of *SiMADS51* Reduced Drought Tolerance in Transgenic *Arabidopsis* Plants

**FIGURE 8:**
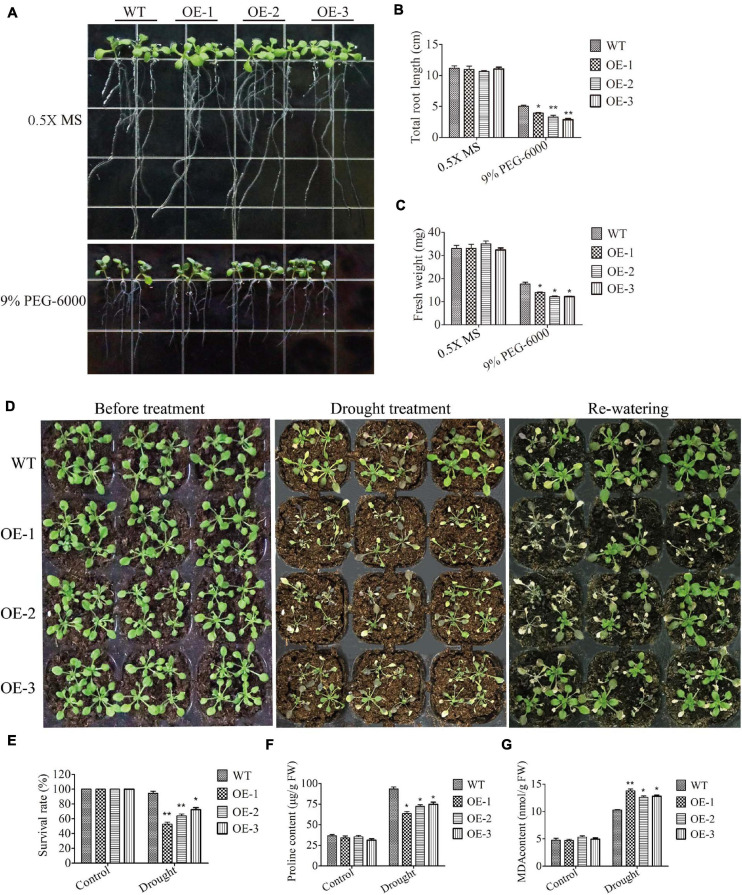
Overexpression of *SiMADS51* reduced drought tolerance in transgenic *Arabidopsis* plants. **(A)** Root length assays of wild-type (WT) and *SiMADS51*-overexpressing plants under normal conditions and PEG-6000 treatment. **(B)** Total root lengths of seedlings. **(C)** Fresh weight of normal and PEG-6000 stressed plants. **(D)** Drought tolerance phenotypes of WT and *SiMADS51* transgenic *Arabidopsis* in soil. Three-week-old seedlings of WT and *SiMADS51*-overexpressing lines were dehydrated for 1 week and then rehydrated for 3 days. **(E)** Survival rate of normal and drought-stressed plants. **(F)** Proline and **(G)** MDA content were detected in WT and OE plants under drought or normal growth condition. Data are presented as the means ± SDs of three independent replicates. The asterisks indicate significant differences between WT and *SiMADS51*-overexpressing lines (*P < 0.05; **P < 0.01; Student’s *t*-test).

To investigate the tolerance of *SiMADS51*-overexpressing lines to drought stress, transgenic *Arabidopsis* seedlings were cultured on 0.5X MS medium supplemented with PEG-6000 and also subjected to drought stress in soil. When grown on 0.5X MS medium, WT and *SiMADS51*-overexpressing (OE-1, OE-2, and OE-3) plants exhibited similar growth status (Figure 8A). However, on 0.5X MS medium supplemented with 9% PEG-6000, the roots of the transgenic line seedlings were shorter than those of the wild-type plants, and plants of the three transgenic lines had lower fresh weights compared to the wild-type plants (Figures 8A–C). When grown in soil, no significant differences in morphology were observed between wild-type plants and the three *SiMADS51*-overexpressing lines prior to drought treatment (Figure 8D). After drought stress treatment, seedlings of the *SiMADS51*-overexpressing lines appeared to be in poorer physical condition compared to the wild-type plants. As can be seen in Figure 8D, the OE line plants were badly wilted and bleached, while the WT plants were only slightly damaged. After re-watering, the survival rate of the wild-type *Arabidopsis* plants was higher than that of the three OE lines (Figure 8E). To study the possible physiological mechanism that explains the decreased drought tolerance in the *SiMADS51*-overexpressing lines, some stress-related physiological indicators were measured for the OE lines and WT plants grown under normal and drought conditions. Under normal growth conditions, there were no significant differences in the proline and MDA contents between the OE and WT plants (Figures 8F,G). However, the proline content in the WT plants was higher than in the OE plants under drought stress conditions (Figure 8F). For MDA, the contents measured in detached leaves from the *SiMADS51* overexpressing lines were higher than in the WT plants under drought stress conditions (Figure 8G). These results indicate that decreased contents of stress-related metabolites and enhanced membrane lipid peroxidation may be the causes of reduced drought tolerance in the *SiMADS51*-overexpressing *Arabidopsis* plants.

### Overexpression of *SiMADS51* Reduces Tolerance to Drought Stress in Transgenic Rice Plants

**FIGURE 9:**
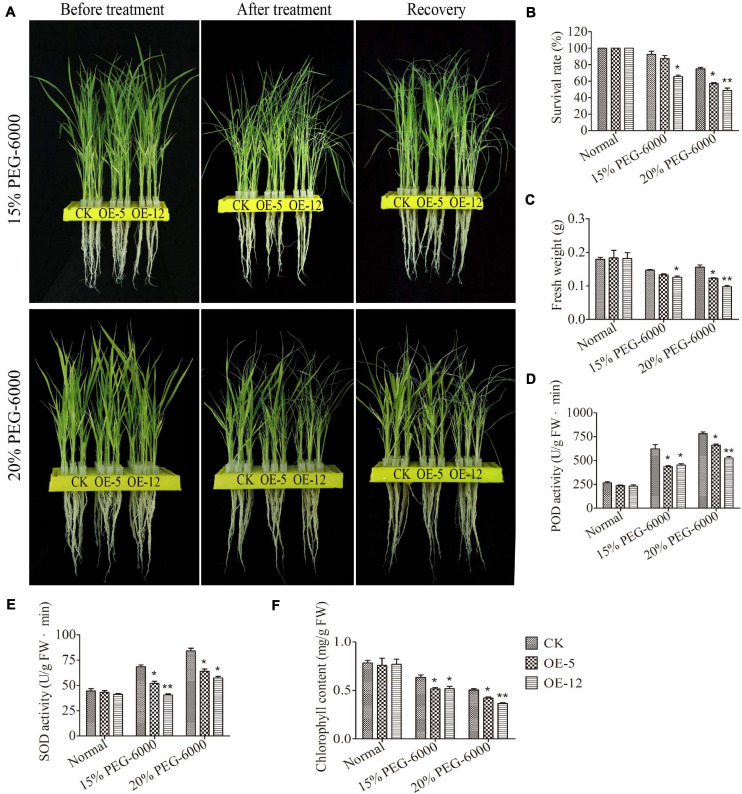
Overexpression of *SiMADS51* reduces tolerance to drought stress in transgenic rice plants. **(A)** Phenotypes of CK and *SiMADS51*-transgenic rice under the treatment of PEG-6000. **(B)** Survival rate and **(C)** fresh weight of normal and PEG-6000 stressed plants. **(D)** POD, and **(E)** SOD activities were measured under PEG-6000 stress and normal growth conditions. **(F)** Chlorophyll content were detected in CK and OE plants under PEG-6000 stress or normal growth condition. Data are presented as the means ± SDs of three independent replicates. The asterisks indicate significant differences between WT and *SiMADS51*-overexpressing lines (*P < 0.05; **P < 0.01; Student’s *t*-test).

To gain further evidence of how *SiMADS51* overexpression reduces plant tolerance to drought stress, we transformed the *SiMADS51* gene into rice, and obtained 19 independent transgenic lines. The relative expression levels of *SiMADS51* gene in OE-5 and OE-12 line plants (T_3_ generation) were higher than those of the other lines (Supplementary Figure 2B). Thus, the transgenic lines OE-5 and OE-12 were selected for further testing. PEG-6000 treatment was used to mimic dehydration stress conditions, and the drought tolerance of the two *SiMADS51*-overexpressing rice lines and control line (CK) plants were assessed at the vegetative growth stage. As shown in Figure 9A, all the seedlings exhibited similar growth status before stress treatment, while exposure to PEG-6000 stress for 1 week led to leaf wilting and even drying. However, in the 15% PEG-6000 stress treatment, leaves of the control plants showed delayed wilting and less curling compared to plants of the two transgenic OE lines (Figure 9A). After returning to normal growth conditions, only 57% and 49% of the transgenic OE-5 and OE-12 line seedlings lines recovered from the 20% PEG-6000 treatment, while 75% of the control seedlings survived (Figure 9B). In addition, the fresh weights of the transgenic line seedlings were less than those of the CK plants (Figure 9C). To investigate the physiological changes that occurred in the *SiMADS51*-OE and CK plants, the chlorophyll content and the POD and SOD activities were measured under drought stress and normal growth conditions. After PEG-6000 treatment, the POD and SOD activities increased significantly in both the *SiMADS51*-OE and CK plants compared with the activities in plants grown under normal conditions (Figures 9D,E). Under drought stress conditions, the POD and SOD activities in *SiMADS51*-OE plants were much lower than in the CK plants (Figures 9D,E). Furthermore, the chlorophyll contents of the *SiMADS51*-OE plants under PEG-6000 stress were also significantly lower than in the CK plants (Figure 9F). These results indicate that under drought stress, the photosynthesis rate and reactive oxygen species (ROS) scavenging capacity of the *SiMADS51*-OE plants were reduced compared to the CK plants, which led to the observed reduction in drought tolerance of the transgenic line plants.

### *SiMADS51* Expression Alters the Transcription of a Group of Abiotic Stress-Related Genes in Rice

**FIGURE 10:**
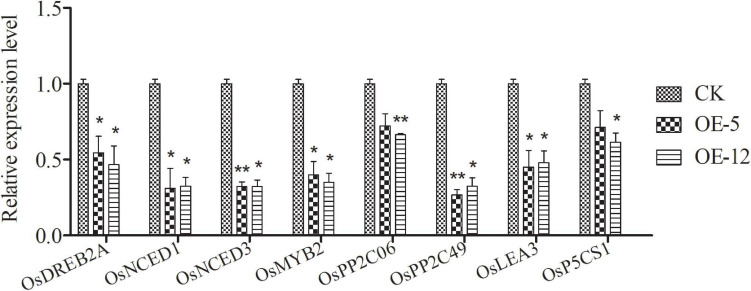
Expression of several stress-related genes in *SiMADS51*-overexpressing plants (OE-5 and OE-12) and control line (CK) under normal condition. The expression of each gene was normalized using the *actin* gene (*LOC_Os03g50885*) as control. Data are presented as the means ± SDs of three independent replicates. Asterisks indicate statistical significance (*P < 0.05; and **P < 0.01; Student’s *t*-test) compared with the corresponding controls.

To explore the possible molecular mechanisms responsible for the reduced drought tolerance in transgenic plants, we examined the expression profiles of several rice genes reported to be related to abiotic stress tolerance. Leaves from normally grown plants were sampled for RT-qPCR analysis. As shown in Figure 10, compared with the control plants, the expression levels of *OsDREB2A* (Dubouzet et al., 2003), *OsMYB2* (Yang et al., 2012), and *OsPP2C06* and *OsPP2C49* (Singh et al., 2010), some signal transduction- and regulation-related genes known to be associated with abiotic stress tolerance, were down-regulated in the transgenic lines. Likewise, the expression level of two late stress-responsive genes, *OsLEA3* (Xiao et al., 2007) and *OsP5CS1* (Xiong et al., 2014), were also down-regulated in the transgenic lines. We also found that the expression of *OsNCED1* and *OsNCED3* (Hwang et al., 2010; Changan et al., 2018), two stress-related genes involved in ABA biosynthesis, was down-regulated in the transgenic plants grown under normal condition. These results indicate that *SiMADS51* may act as a negative regulator to inhibit the expression of abiotic stress-related genes, thereby impairing drought tolerance in plants.

## Discussion

Foxtail millet (*Setaria italica* L.) is one of the oldest cultivated crops in China (Jia et al., 2013). With the development of high quality varieties and the improvement of cultivation techniques in recent years, improving the quality and production efficiency of millet will become a major research focus. Previous studies have shown that MADS-box TFs play important roles in plant growth, development, and the abiotic stress response (Honma and Goto, 2001; Becker and Theißen, 2003; Lee et al., 2008b; Khong et al., 2015). Therefore, the *MADS-box* gene family could be a key resource for promoting transgenic crops and traditional breeding to increase yield.

In this paper, a total of 72 *MADS-box* genes were identified in the foxtail millet genome (Table 1). In comparison with previously published studies, the number of *MADS-box* genes vary between monocotyledons and dicotyledons; for example, rice (*Oryza sativa* L.) (75), maize (*Zea mays* L.) (75), and *Sorghum bicolor* (65) (Arora et al., 2007; Zhao et al., 2011), all have fewer *MADS-box* genes than *Arabidopsis* (109), potato (*Solanum tuberosum*) (156), and tomato (*Solanum lycopersicum*) (131) (Gao et al., 2018; Wang et al., 2019). This suggests that monocotyledonous plants might have lost *MADS-box* genes during evolution. To examine the gain and loss of *MADS-box* genes in foxtail millet, a phylogenetic tree was constructed using the amino acid sequences of MADS-box proteins from foxtail millet, *Setaria viridis*, *Arabidopsis*, potato, and rice. As can be seen in Figure 1 and Supplementary Table 1, the number of Type I *MADS-box* genes in *Arabidopsis* (63) and potato (116) was much higher than that in foxtail millet (43), rice (45), and *Setaria viridis* (43), while, the number of Type II *MADS-box* genes was similar in the five species. Therefore, we speculated that during the evolution of monocotyledons, the loss of *MADS-box* genes occurred mainly in the Type I clade. In foxtail millet, the Type II *MADS-box* genes could be divided into MIKC^*c*^ and MIKC^∗^ clades, and the MIKC^*c*^ clade is further divided into 12 smaller subclades. In the OsMADS32-like subclade, the *MADS-box* genes are present in related monocot species but not in *Arabidopsis* and potato, which suggests that these may have evolved after the divergence of monocots and dicots. In the AGL17, AGL12, Bsister, and PI (GLO) subclades, there are more MADS-box genes from rice, *Setaria viridis*, and foxtail millet than that from *Arabidopsis* and potato, suggesting these subclades expanded in during the evolution of monocots.

The plant *MADS-box* gene family appears to have expanded mainly through tandem duplication events (Airoldi and Davies, 2012). It was previously reported that different groups of *MADS-box* genes underwent expansion in different plant species, such as M-type genes in rice, *SVP*-like genes in cotton, and *AGL1*7-like and *Bsister*-like genes in wheat (*Triticum aestivum* L.) (Arora et al., 2007; Nardeli et al., 2018; Schilling et al., 2020). Our analysis revealed that the amplification of foxtail millet *MADS-box* genes was due to segmental duplication alone (Figure 2 and Table 2). Also, the Ka/Ks ratios indicate that the *SiMADS-box* genes mainly experienced purifying selection (Ka/Ks ratio < 1) during evolution (Zhang et al., 2006). This evidence indicates that the expansion (and contraction) of the foxtail millet *MADS-box* gene family might be to allow it to adapt to changes in the environment, thereby contributing to its wider distribution (Theißen et al., 2018). In some cases, neofunctionalization and subfunctionalization might be involved in gene duplication (Irish and Amy, 2005).

It is known that the structural diversity of genes drives the evolution of multigene families. Studies have shown that intron loss and insertion mutations are common during the evolution of plant *MADS-box* genes (Wei et al., 2015; Gao et al., 2018). In this study, we found that the number of introns in the *SiMADS-box* genes varied greatly (ranging from 0 to 11), most of the M-type *MADS-box* genes in foxtail millet had no introns, with a few exceptions, while the MIKC^∗^ genes had 9 to 11 introns, the most in any group of *SiMADS-box* genes, and was more than in other genes in the MIKC^*c*^ subgroup (Table 1 and Figure 2C). Also, the protein motifs present in proteins the same family were not identical (Figure 2B). These differences among *MADS-box* genes in the same subgroup suggest that the loss or acquisition of introns may be a pattern of *SiMADS-box* gene evolution, and could be a major contributing factor to the functional diversity of the foxtail millet MADS-box family. Despite the differences, most of the *SiMADS-box* genes in the same group had similar intron-exon arrangements and the predicted proteins had a similar complement of motifs (Figures 2A–C). Similar results have been reported in monocots species such as rice (Arora et al., 2007) and wheat (Schilling et al., 2020), which imply evolutionary conservation within the MADS-box family.

*Cis-*regulatory elements play an important role in the regulation of plant gene expression; therefore, we conducted a systematic analysis of the CREs present in the promoter regions of the *SiMADS*-box genes. A large number of GA, ABA, defense, and dehydration responsive elements were identified in the *SiMADS-box* genes promoter region, which suggests that *SiMADS-box* genes might be regulated by various phytohormones, abiotic and biotic stresses. In addition, combined with our transcriptome sequencing data, we identified 10 foxtail millet *MADS-box* genes belonging to eight of MIKC-type subgroups in which expression was induced by drought stress. The promoter regions of these 10 *SiMADS-box* genes contain several stress-related *cis*-elements such as ABREs, GAREs, MYB, MYC, and W box (Table 3), indicating that the expression of *SiMADS-box* genes can respond to multiple environmental cues. This is supported by further RT-qPCR analysis showing that the 10 *SiMADS-box* genes were induced significantly by PEG-6000, NaCl, ABA, and GA treatments (Figure 6). Moreover, tissue-specific expression patterns for the 10 *SiMADS-box* genes varied considerably in four plant organs (Figure 5), indicating the functional diversity of the SiMADS-box proteins. This knowledge could establish a foundation for further functional characterization of *MADS-box* gene family members in foxtail millet and other important crops.

In the present study, we observed that transgenic *Arabidopsis* and rice plants overexpressing *SiMADS51* showed reduced drought stress tolerance as indicated by the lower survival rates and poorer growth characteristics under drought stress conditions (Figures 7–9), which demonstrated that SiMADS51 is a negative regulator of drought stress tolerance in plants. Previous studies have shown that *AtMADS028* (XAL1/AGL12; Tapia-López et al., 2008), an *Arabidopsis* gene homologous to *SiMADS51*, plays a role in regulating the root meristem, cell proliferation, and the flowering transition. The *SiMADS51*-homologous gene in rice, *OsMADS61* (*OsMADS26*; Khong et al., 2015), was found to negatively regulate resistance to pathogens and drought tolerance and had no significant impact on plant development. Homologous genes play different roles in monocotyledons and dicotyledons, which further illustrates the functional diversity of *MADS-box* genes. The mechanisms that underly the impaired drought tolerance in the *SiMADS51*-transgenic plants can be partially explained by the measured changes in several physiological and biochemical parameters. Generally, free proline is thought to be a compatible osmolyte that facilitates osmo-regulation, and it can help plants tolerate osmotic stress (Kochhar and Kochhar, 2005). The activities of POD and SOD can play a key role in protecting cell membranes against free radical attack and minimizing oxidative damage (Shah and Nahakpam, 2012). The chlorophyll content and MDA level can also be used as indicators of the degree of cellular injury (Gunes et al., 2011; Hong et al., 2016). In our study, we found that transgenic plants overexpressing *SiMADS51* had higher MDA contents than did the control plants, while the free proline levels and chlorophyll contents and the POD and SOD activities in the *SiMADS51*-overexpressio plants were lower than those of the control plants under drought treatment. All of these results suggest that overexpression of *SiMADS51* reduced the tolerance of seedlings to drought stress, resulting in increased membrane permeability, reduced activity of peroxidases, and decreased photosynthesis. In addition, we also noticed that overexpression of *SiMADS51* in transgenic rice seedlings resulted in a decrease in the expression of a group of stress-responsive genes (Figure 10), including *OsPP2C06*, *OsPP2C49* (Singh et al., 2010), *OsDREB2a* (Dubouzet et al., 2003), *OsMYB2* (Yang et al., 2012), *OsLEA3* (Xiao et al., 2007), *OsP5CS1* (Xiong et al., 2014), *OsNCED1* (Changan et al., 2018), and *OsNCED3* (Hwang et al., 2010), which have been previously reported to be involved in the stress response. Hence, we can speculate that the decrease in plant stress tolerance may be due to SiMADS51 directly or indirectly inhibiting the expression of these genes. Nevertheless, it is not clear how *SiMADS51* affects the function of other genes to reduce plant drought tolerance. Even though the expression of *SiMADS51* is significantly induced by exogenous ABA, it is unknown whether its function is dependent on the ABA-mediated pathway. Moreover, functional analysis using knockout mutants is necessary to determine whether *SiMADS51* is required for drought tolerance in foxtail millet. Therefore, further studies will be needed to clarify the mechanism(s) by which *SiMADS51* regulates the response to abiotic stresses.

## Conclusion

In this study, we report the first systematic analysis of the foxtail millet (*Setaria italica* L.) MADS-box TF family. A total of 72 *MADS-box* genes including 29 Type I and 43 Type II genes, were identified in the foxtail millet genome. The phylogenetic relationships, chromosomal distribution, conserved motifs, gene structures, and *cis*-acting elements of the 72 foxtail millet *MADS-box* genes were characterized. Furthermore, the expression patterns of 10 foxtail millet *MADS-box* genes that are upregulated in response to drought were analyzed in different organs in response to different abiotic stresses. Because the *SiMADS51* genes was found to be strongly induced by drought stress, the function of *SiMADS51* gene was assessed by expression in the model plants *Arabidopsis* and rice (*Oryza sativa* L.). Finally, *SiMADS51* was shown to be involved in the negative regulation of drought stress. Our results provide evidence of the relationship between foxtail millet *MADS-box* genes and abiotic stresses; therefore, it may be possible to use these genes for both genetically modified crops and in traditional foxtail millet breeding programs.

## Data Availability Statement

The original contributions presented in the study are included in the article/Supplementary Material, further inquiries can be directed to the corresponding authors.

## Author Contributions

D-HM and Y-ZM conceived and designed the experiments. Z-SX edited the manuscript. WZ performed the experiments and wrote the first draft. L-LZ conducted the bioinformatic work and revised the manuscript. LF and H-XP contributed to data analysis. All authors have read and agreed to the published version of the manuscript.

## Conflict of Interest

The authors declare that the research was conducted in the absence of any commercial or financial relationships that could be construed as a potential conflict of interest.

## Footnotes

1 http://planttfdb.cbi.pku.edu.cn/

2 https://phytozome.jgi.doe.gov/pz/portal.html/

3 http://pfam.xfam.org/

4 https://web.expasy.org/protparam/

5 http://meme-suite.org/tools/meme

6 http://gsds.gao-lab.org/

7 https://www.dna.affrc.go.jp/PLACE/?action=newplace

8 https://www.ebi.ac.uk/gxa/home

## Supplementary Material

Supplementary Figure 1 Subcellular localization of *SiMADS-box* proteins in rice protoplasts. Results were visualized with confocal microscopy 16 h after transformation. Scale bars = 10 μm.

Supplementary Figure 2 Relative transcript level of *SiMADS51* gene in transgenic *Arabidopsis* and rice lines. **(A)** Relative transcript level of *SiMADS51* gene in 7 transgenic *Arabidopsis* lines. **(B)** Relative transcript level of *SiMADS51* gene in 19 transgenic rice lines.

Supplementary Figure 3 Overexpression of *SiMADS5*1 reduces tolerance to drought stress in transgenic rice plants.

Supplementary Table 1 The 488 *MADS-box* genes identified from *Arabidopsis*, rice, foxtail millet, potato, and *Setaria viridis* in this study.

Supplementary Table 2 The amino acid sequences of 20 putative motifs.

Supplementary Table 3 All the *cis*-regulatory elements of 72 *SiMADS-box* gene promoters predicted by New PLACE online tool.

Supplementary Table 4 Twenty-five *SiMADS-box* genes that showed a transcriptional response to drought stress were identified from RNA-seq data of drought-treated foxtail millet seedlings.

Supplementary Table 5 The FPKM (fragments per kilobase of transcript per million mapped reads) values of different tissues of 10 *SiMADS-box* genes.

Supplementary Table 6 Primers used in this study.

## Abbreviations

ABA: abscisic acid
ABRE: ABA-responsive elements
CDS: coding sequence length
FPKM: fragments per kilobase of transcript per million mapped reads
FW: fresh weight
GA: gibberellic acid
GARE: GA responsive elements
Ka: non-synonymous substitution rate
Ks: synonymous substitution rate
LTRE: low-temperature responsive element
MDA: malonaldehyde
NCBI: National Center for Biotechnology Information
MeJA: methyl jasmonate
POD: peroxidase
RT-PCR: reverse transcription-PCR
qPCR: quantitative real-time-PCR
RT-qPCR: quantitative real-time RT-PCR
SA: salicylic acid
SOD: superoxide dismutase
TF: transcription factor.
