# Supplementary figures and images for: Genome-Wide Analysis of MADS-Box Genes in Foxtail Millet (Setaria italica L.) and Functional Assessment of the Role of SiMADS51 in the Drought Stress Response
Source: Front Plant Sci. 2021 Jun 28;12:659474. doi: 10.3389/fpls.2021.659474 (PMC8273297; doi:10.3389/fpls.2021.659474)

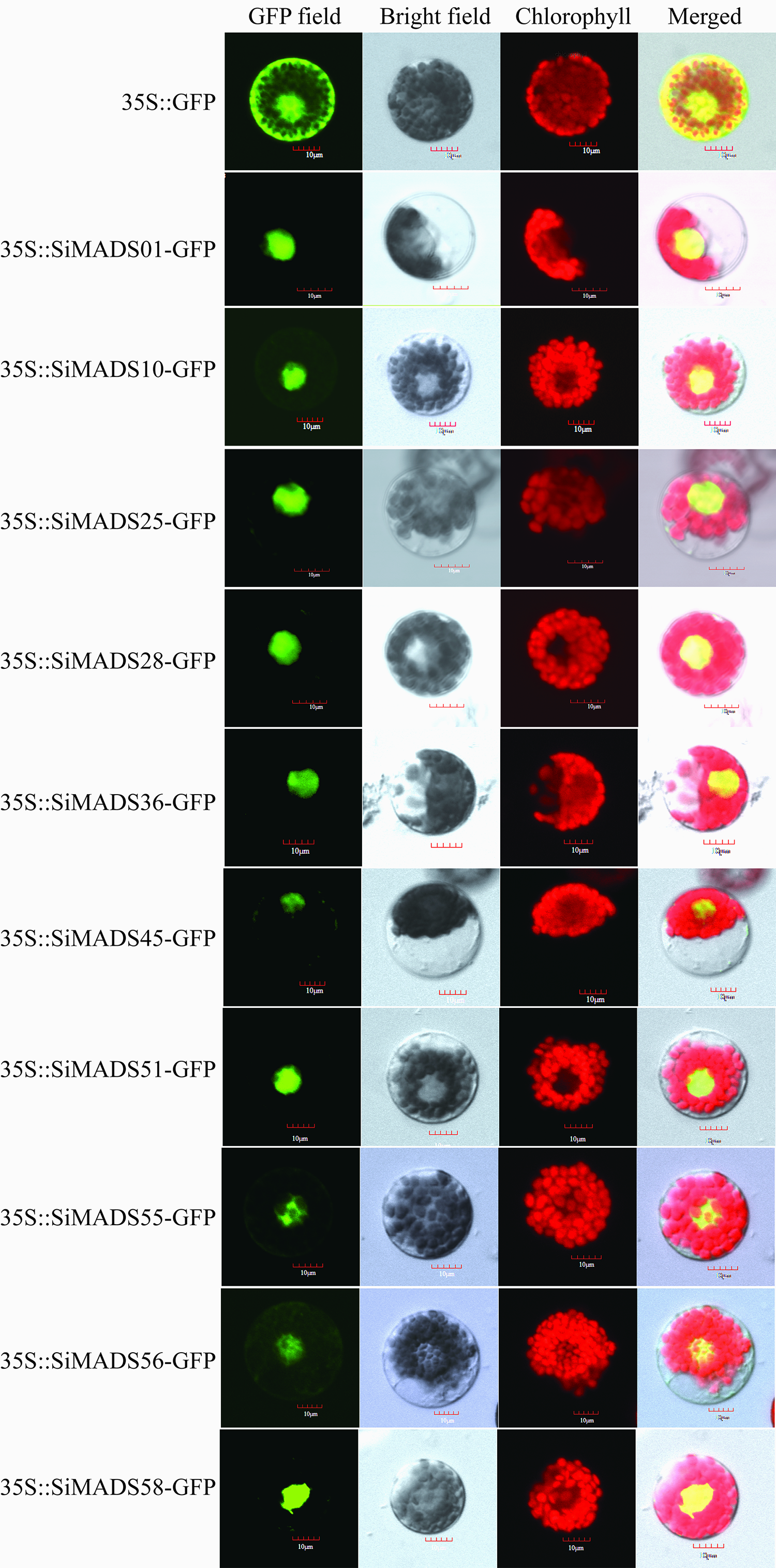

Supplement: Supplementary Figure 1 — Subcellular localization of SiMADS-box proteins in rice protoplasts. Results were visualized with confocal microscopy 16 h after transformation. Scale bars = 10 μm. [file Image_1.TIF]

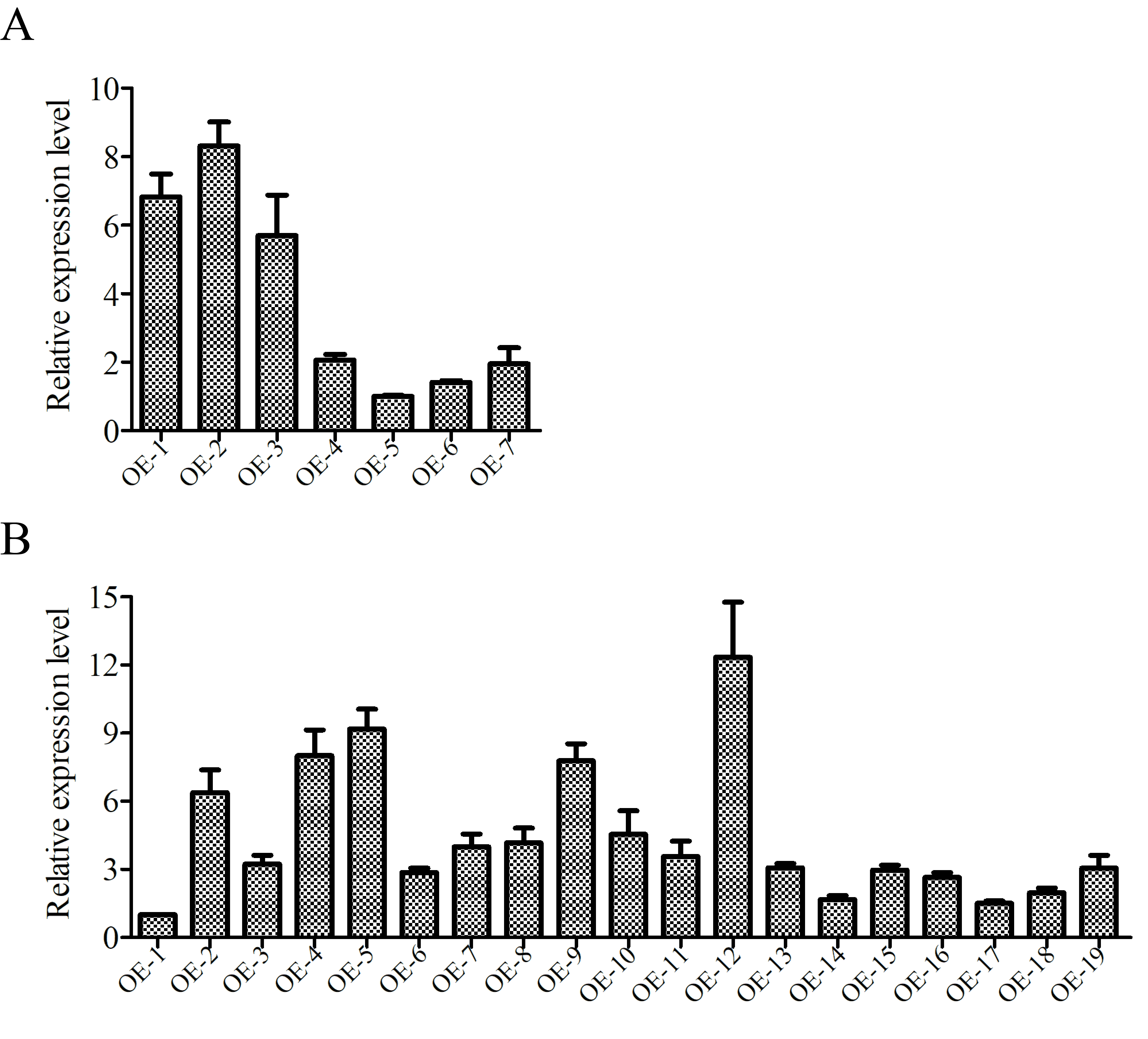

Supplement: Supplementary Figure 2 — Relative transcript level of SiMADS51 gene in transgenic Arabidopsis and rice lines. (A) Relative transcript level of SiMADS51 gene in 7 transgenic Arabidopsis lines. (B) Relative transcript level of SiMADS51 gene in 19 transgenic rice lines. [file Image_2.TIF]

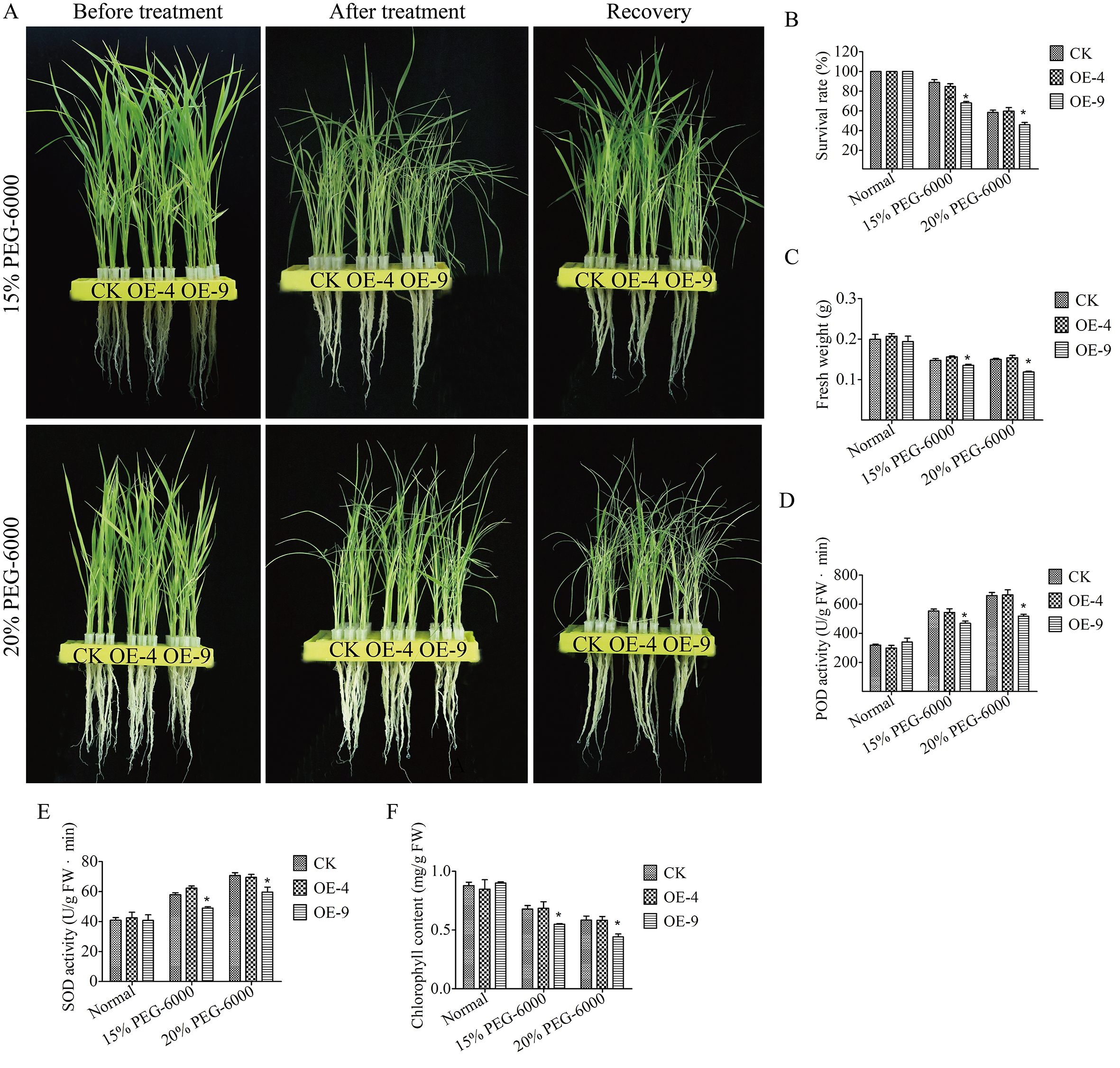

Supplement: Supplementary Figure 3 — Overexpression of SiMADS51 reduces tolerance to drought stress in transgenic rice plants. [file Image_3.TIF]
